# Analyzing the Clinical Potential of Cold Atmospheric Plasma in Dentistry as an Alternative to Antibiotic Therapy. Comment on Gross et al. Guided Plasma Application in Dentistry—An Alternative to Antibiotic Therapy. *Antibiotics* 2024, *13*, 735

**DOI:** 10.3390/antibiotics14030272

**Published:** 2025-03-07

**Authors:** Thibault Canceill, Cristina Canal, Antoine Dubuc, Nofel Merbahi, Sarah Cousty

**Affiliations:** 1 Biomaterials, Biomechanics and Tissue Engineering Group, Materials Science and Engineering Department, Research Center for Biomedical Engineering, Universitat Politècnica de Catalunya·BarcelonaTECH (UPC), Escola d’Enginyeria Barcelona Est (EEBE), C/Eduard Maristany 14, 08019 Barcelona, Spain; cristina.canal@upc.edu; 2 InCOMM (Intestine ClinicOmics Microbiota & Metabolism), UMR1297 Inserm/Université Toulouse III, French Institute of Metabolic and Cardiovascular Diseases (i2MC), 1 Avenue Jean Poulhès, 31400 Toulouse, France; 3 Laplace, Université de Toulouse, CNRS, INPT, UPS, Toulouse, 118 Route de Narbonne, 31062 Toulouse, France; nofel.merbahi@laplace.univ-tlse.fr (N.M.); sarah.cousty@univ-tlse3.fr (S.C.); 4 Toulouse Universitary Hospital, Dental and Oral Medicine Department, 3 Chemin des Maraichers, 31400 Toulouse, France; antoine.dubuc@univ-tlse3.fr; 5 Barcelona Research Center in Multiscale Science and Engineering, UPC, 08019 Barcelona, Spain; 6 I2SO Institut de Simulation en Santé Orale—Institute of Simulation in Oral Healthcare, Paul Sabatier Toulouse III University, 3 Chemin des Maraichers, 31400 Toulouse, France

**Keywords:** plasma medicine, antibiotic resistance, commentary

The study by Gross et al. explores the potential of cold atmospheric plasma (CAP) as an alternative to antibiotics in dentistry [1], presenting it as a promising tool to address antimicrobial resistance. This represents a major global public health challenge [2,3], with alarming projections estimating that it could cause up to 10 million deaths annually by 2050 if no effective solutions are implemented [4]. Antibiotic resistance extends far beyond the dental field. Resistance is driven not only by improper prescriptions but also by environmental factors such as antibiotic contamination in water and soil [5]. Thus, public health measures might yield greater benefits than introducing new antimicrobial technologies for localized dental applications.

Gross et al. demonstrate the effective elimination of both aerobic and anaerobic bacteria without inducing resistance or significant temperature increases. The AmbiJet device delivers CAP directly to the target site, thus allowing localized treatment with minimal collateral tissue damage. However, the conclusion that CAP can be suitable for the prevention of periimplantitis appears somewhat premature given the scope of the study. The in vitro experimental setting and the limited range of bacterial strains tested do not fully represent the clinical challenges of diverse patient populations. The study does not address individual variability in oral microbiomes or the potential long-term effects of CAP on healthy tissues, which are critical factors for clinical application. Although the study mentions the impact of CAP on biofilms, more robust evidence is needed to confirm its efficacy against established and multilayered biofilm structures under real-world conditions.

Another question concerns the rationale for applying this technology directly to the implant as proposed in the experimental model of the study. This model is not very common in clinics. The gingiva is more concerned with plasma therapy than bone, and in clinical practice, topical antibiotics are not used on bone tissue, as systemic delivery remains the gold standard in most dental and medical settings. Moreover, they are never used in the prevention of peri-implantitis, so the plasma system proposed here cannot be presented as “an alternative to antibiotic therapy”. In countries like France, even the use of prophylactic antibiotics during routine implant placement is not recommended, provided that practitioners adhere to established guidelines. Consequently, the development of alternative antibacterial systems, such as CAP, may not be as critical as improving compliance with existing antibiotic stewardship recommendations.

The article lacks a detailed evaluation of CAP’s impact on oral mucosa and other non-target tissues, which are essential for assessing clinical safety. Future research should focus on addressing these gaps, and long-term in vivo studies are necessary to evaluate the effects of CAP on oral tissues and microbiomes. Expanding the spectrum of pathogens and exploring its efficacy against complex biofilms will provide more comprehensive insights into its therapeutic potential. Real-world studies should also consider patient comfort, procedural feasibility, and potential barriers to implementation, such as device portability, integration into existing dental workflows and costs. The initial investment required for technologies such as cold atmospheric plasma may pose a significant barrier, particularly for smaller practices or those operating in economically disadvantaged areas. While the AmbiJet device demonstrates promising efficacy, its widespread implementation will likely depend on its affordability and cost-effectiveness relative to existing dental treatments.

Despite these considerations, CAP does offer one significant advantage: as an anti-infective agent, it does not induce more resistance in micro-organisms. However, in the field of dentistry, resistance to antibiotics remains rare (for example, less than 3% in German private practices regarding penicillin and aminopenicillin [6]), and there is currently no widespread evidence to suggest that new systems like CAP are urgently needed to address this issue.

To conclude, the findings presented by Gross et al. undoubtedly represent an interesting step forward in exploring the possibility of using cold atmospheric plasmas in dentistry. However, it is essential to approach these results with caution. While the potential of CAP in combating antibiotic resistance is promising, its clinical adoption will require rigorous, long-term research to ensure its safety, efficacy, and practicality.
